# Supplementary material for: Piezoelectric truss metamaterials: data-driven design and additive manufacturing
Source: NPJ Metamater. 2025 Dec 3;1(1):9. doi: 10.1038/s44455-025-00009-2 (PMC12675293; doi:10.1038/s44455-025-00009-2)
Supplement: Supplementary file 1 — Supplementary information [file 44455_2025_9_MOESM1_ESM.pdf]

Piezoelectric truss metamaterials: data-driven design and additive manufacturing

Supporting Information

Saurav Sharma[#1,2,3], Satya K. Ammu[#1], Prakash Thakolkaran[2], Jovana Jovanova[*,3], Kunal Masania[*,1], and Siddhant Kumar[*,2]

[1]Shaping Matter Lab, Faculty of Aerospace Engineering, Delft University of Technology, 2629 HS, Delft, The Netherlands.
[2]Department of Materials Science and Engineering, Faculty of Mechanical Engineering, Delft University of Technology, 2628 CD, Delft, The Netherlands.
[3]Department of Maritime and Transport Technology, Faculty of Mechanical Engineering, Delft University of Technology, 2628 CD, Delft, The Netherlands.
[*]J.Jovanova@tudelft.nl, K.Masania@tudelft.nl, Sid.Kumar@tudelft.nl

Contents

1 Numerical homogenization **1**
2 ML-based design optimization **3**
2.1 Design space I 3
2.2 Design space II 3
3 Dataset exploration **4**
4 Additional examples **5**
5 Principal component analysis (PCA) **5**
References **11**

1 Numerical homogenization

To compute the effective electromechanical properties of the piezoelectric metamaterials, we developed an in-house code based on Bernoulli-Euler beam elements. We employ two-noded elements with seven degrees of freedom (dofs) per node, including six mechanical dofs and electric potential as the electrical dof. We extend the asymptotic homogenization framework developed by Zhang et al.[1] for elastic properties to compute the effective electromechanical properties of the piezoelectric metamaterials. After assembling the stiffness matrix of the unit cell, the global FE equation can be written as

$$\boldsymbol{K}\boldsymbol{U} = \boldsymbol{f}, \tag{1}$$

where $\boldsymbol{K}$ is the global stiffness matrix, $\boldsymbol{U}$ is the displacement vector, and $\boldsymbol{f}$ is the force vector corresponding to mechanical and electric dofs. The nodal dofs are ordered as $\{u_x\ u_y\ u_z\ \theta_x\ \theta_y\ \theta_z\ \phi\}^T$, where u, and θ are the displacement and rotations, and ϕ is the nodal electric potential. Since the poling direction is considered to be along Z axis, the piezoelectric tensor $\boldsymbol{e}^{\text{base}}$ is first transformed to local coordinates (x, y, z) using the transformation matrix $\boldsymbol{R}$ written as

$$\boldsymbol{R} = \begin{bmatrix} \beta_{Xx} & \beta_{Yx} & \beta_{Zx} \\ \beta_{Xy} & \beta_{Yy} & \beta_{Zy} \\ \beta_{Zx} & \beta_{Zy} & \beta_{Zz} \end{bmatrix}, \tag{2}$$

where β_{Xx}, β_{Yx}, and β_{Zx} are the direction cosines of the x-axis, with respect to global X, Y, and Z axes, respectively, and the similar notation is followed for the second and third rows of the matrix. The transformed $\boldsymbol{e}^{\text{base}}$ is then used to compute the electromechanical FE stiffness matrix in the struts' local coordinates xyz.

Since all the unit cells generated in both the datasets have periodic geometry, the corners, opposing faces, and parallel edges can be coupled using a transformation matrix $\boldsymbol{T}$ as $\boldsymbol{U} = \boldsymbol{T}\tilde{\boldsymbol{U}}$. Where $\tilde{\boldsymbol{U}}$ is the reduced displacement vector under periodic boundary conditions (PBCs). The expanded matrix form of this transformation can be written as[2,3]

$$\begin{bmatrix} U_{C1} \\ U_{C2} \\ U_{C3} \\ U_{C4} \\ U_{C5} \\ U_{C6} \\ U_{C7} \\ U_{C8} \\ U_{E1} \\ U_{E2} \\ U_{E3} \\ U_{E4} \\ U_{E5} \\ U_{E6} \\ U_{E7} \\ U_{E8} \\ U_{E9} \\ U_{E10} \\ U_{E11} \\ U_{E12} \\ U_{F1} \\ U_{F2} \\ U_{F3} \\ U_{F4} \\ U_{F5} \\ U_{F6} \\ U_{int} \end{bmatrix}_{n\times 1} = \begin{bmatrix} I & 0 & 0 & 0 & 0 & 0 & 0 & 0 \\ I & 0 & 0 & 0 & 0 & 0 & 0 & 0 \\ I & 0 & 0 & 0 & 0 & 0 & 0 & 0 \\ I & 0 & 0 & 0 & 0 & 0 & 0 & 0 \\ I & 0 & 0 & 0 & 0 & 0 & 0 & 0 \\ I & 0 & 0 & 0 & 0 & 0 & 0 & 0 \\ I & 0 & 0 & 0 & 0 & 0 & 0 & 0 \\ I & 0 & 0 & 0 & 0 & 0 & 0 & 0 \\ 0 & I & 0 & 0 & 0 & 0 & 0 & 0 \\ 0 & 0 & I & 0 & 0 & 0 & 0 & 0 \\ 0 & 0 & 0 & I & 0 & 0 & 0 & 0 \\ 0 & I & 0 & 0 & 0 & 0 & 0 & 0 \\ 0 & I & 0 & 0 & 0 & 0 & 0 & 0 \\ 0 & I & 0 & 0 & 0 & 0 & 0 & 0 \\ 0 & 0 & I & 0 & 0 & 0 & 0 & 0 \\ 0 & 0 & I & 0 & 0 & 0 & 0 & 0 \\ 0 & 0 & I & 0 & 0 & 0 & 0 & 0 \\ 0 & 0 & 0 & I & 0 & 0 & 0 & 0 \\ 0 & 0 & 0 & I & 0 & 0 & 0 & 0 \\ 0 & 0 & 0 & I & 0 & 0 & 0 & 0 \\ 0 & 0 & 0 & 0 & I & 0 & 0 & 0 \\ 0 & 0 & 0 & 0 & 0 & I & 0 & 0 \\ 0 & 0 & 0 & 0 & 0 & 0 & I & 0 \\ 0 & 0 & 0 & 0 & I & 0 & 0 & 0 \\ 0 & 0 & 0 & 0 & 0 & I & 0 & 0 \\ 0 & 0 & 0 & 0 & 0 & 0 & I & 0 \\ 0 & 0 & 0 & 0 & 0 & 0 & 0 & I \end{bmatrix}_{n\times(n-m)} \begin{bmatrix} U_{C1} \\ U_{E1} \\ U_{E2} \\ U_{E3} \\ U_{F1} \\ U_{F2} \\ U_{F3} \\ U_{int} \end{bmatrix}_{(n-m)\times 1}, \tag{3}$$

where n is the total number of dofs, m is the number of master dofs and $(n-m)$ is the number of coupled dofs. $U_{C_1} - U_{C_8}$ are the dofs of the nodes at corners $C_1 - C_8$, $U_{E_1} - U_{E_{12}}$ are the dofs of the nodes at the edges $E_1 - E_{12}$, $U_{F_1} - U_{F_6}$ are the dofs of the nodes at faces $F_1 - F_6$, and U_{int} are the dofs of rest of the nodes, i.e., interior nodes. PBCs are applied by coupling all corner nodes to node C_1, parallel edges to their corresponding reference edge among E_1, E_2, and E_3, and the opposing faces to the reference faces among F_1, F_2, and F_3. The reference corners, edges, and faces are as shown in Figure S1.

Let $\mathbb{C}$ denote the matrix on the right side of Eq. 3 of the main article, which contains homogenized elastic, piezoelectric, and electrical stiffness coefficients. Its components can be computed as

$$\mathbb{C}_{ijkl} = \frac{1}{V}(\boldsymbol{\chi}^{0(ij)} - \tilde{\boldsymbol{\chi}}^{ij})^T(\boldsymbol{f}^{kl} - \boldsymbol{f}^{*kl}), \tag{4}$$

where V is the volume of the unit cell, χ, and $\boldsymbol{f}$ are the nodal displacements and internal force vectors. $\boldsymbol{\chi}^{0(ij)}$ are the initial displacements/electric potential, equivalent to unit strain/electric field applied to the nodes. To obtain

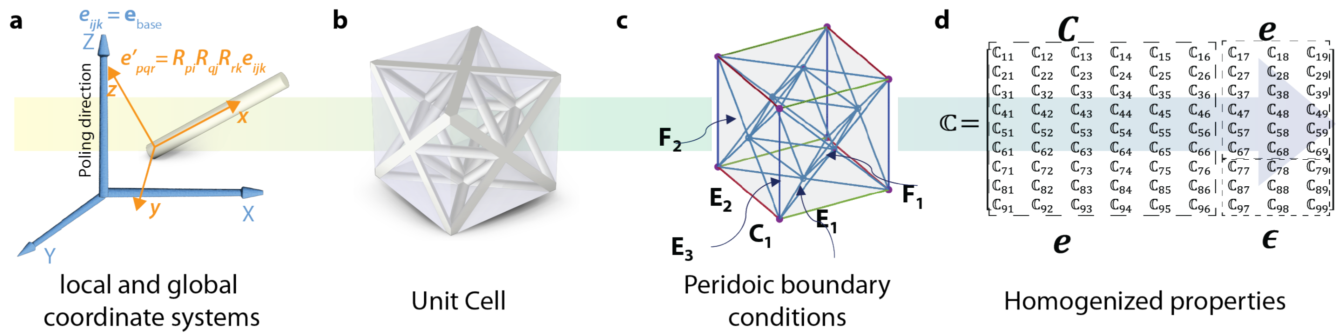

Figure S1. **Schematic representation of the homogenization process. a-b.** The unit cell is first discretized using 1D Bernoulli-Euler elements. **c.** The exterior nodes are categorized into corner, face, and edge nodes, C_1, E_1, E_2, E_3, and F_1, F_2, F_3 being the reference corner, edges, and faces. **d.** Finally, using the asymptotic homogenization approach, the homogenized electromechanical property tensor is computed.

the effective properties, three solution steps are carried out in the following sequence: 1) $\boldsymbol{\chi}^{0(ij)}$ are applied to all the nodes in the unit cell and the generated internal nodal forces are computed as $\boldsymbol{f}^{kl} = \boldsymbol{K}\boldsymbol{\chi}^{0(ij)}$, 2) this nodal force vector is applied under PBCs to compute $\boldsymbol{\chi}^{ij} = \tilde{\boldsymbol{K}}^{-1}\tilde{\boldsymbol{f}}^{kl}$, where $\tilde{\boldsymbol{K}} = \boldsymbol{T}^T\boldsymbol{K}\boldsymbol{T}$, and finally, 3) the computed periodic displacement vector is applied to the system without periodic boundary conditions to compute $\boldsymbol{f}^{*(kl)} = \boldsymbol{K}\tilde{X}^{ij}$. Effective coefficients are then computed by replacing these vectors into eq. (4). The FE homogenization gives the piezoelectric tensor in the stress charge form, which can be used to compute strain charge coefficients, $\boldsymbol{d}$ as

$$\boldsymbol{d} = \boldsymbol{e}.\boldsymbol{S}, \tag{5}$$

where $\boldsymbol{S} = \boldsymbol{C}^{-1}$ is the compliance matrix in Voigt notation.

2 ML-based design optimization

2.1 Design space I

The machine learning models and optimization procedures were trained using specific hyperparameters as outlined in Table S1. The optimization process begins by selecting the S best initial guesses from the dataset as starting points. These input features are concatenated and passed through the neural network to predict the corresponding piezoelectric response. To compute the objective, the predicted piezoelectric matrix is unnormalized to align with the correct physical scales.

The optimization framework employs Gumbel softmax to convert categorical variables—such as lattice types and the number of tessellations—into discrete one-hot encoded representations. Throughout the optimization, the feature set is iteratively updated using the Adam optimizer. The stretch-limiting constraints for the design parameters $\boldsymbol{U}_I$ and $\boldsymbol{V}_I$, are applied as a pre-processing step at each iteration before the property prediction and gradient-based optimization step.

2.2 Design space II

The specific hyperparameters of the second ML framework and the optimization framework are outlined in Table S2. The optimization process involves adjusting the latent variable $\boldsymbol{z}$ to meet specific performance criteria. Initially, we choose a set of S initial designs from the training dataset that perform the best for the objective we aim to optimize. Then, the structural data is encoded into the latent space using the encoder. Since the encoding process is stochastic we use Q different random seeds to encode the initial guesses into the latent space. The resulting latent representations are used as the starting points of the optimization.

At each step, we decode the current latent representation and re-encode it back, analogously to the original optimization procedure[5]. From the re-encoded structure, we predict the piezoelectric matrix components and compute the objective. The optimization process adjusts the latent variables using the Adam optimizer[4], seeking to

Table S1. List of parameters used for the first ML frameworks' training and design optimization protocols.

Parameter	Notation	Value
ML framework #1 hyperparameters:		
Feature scaling	–	none, min-max-scaling
Input dimension forward NN	–	46
Hidden dimensions	–	1024, 1024, 1024, 1024, 1024, 1024
Activation function hidden layer	–	Leaky ReLU
Output dimension	–	18
Learning rate	–	0.001
Batch size	–	8096
Number of epochs	–	300
Optimizer	–	Adam[4]
Train/Test split	–	0.99/0.01
Optimization setup hyperparameters:		
Magnitude regularization parameter	λ_1	0.3
Shrinkage regularization parameter	λ_2	0.3
Stretch scaling	α	0.5
Number of guesses	S	150
Number of epochs	E	10000
Optimizer learning rate	–	0.01
Optimizer	–	Adam[4]

enhance the designs' performance relative to the specified criteria. Figures S2**a** and **b** show the parity plots for the property predictors of dataset I and II, respectively.

3 Dataset exploration

Both the datasets used in this study consist of a wide range of unit cells with different anisotropies of piezoelectric tensor $\boldsymbol{e}$. While dataset I provides the full non-zero piezoelectric tensor, dataset II provides a broader range of topologies within the constraints of a cubic symmetry and hence only five original non-zero piezoelectric coefficients. Figure S3**a** and **b** show the unit cell generation process from a given set of parameters for dataset I and dataset II, respectively.

Figure S4 shows the pair-plots of all 18 coefficients in the dataset I. While a vast landscape of property combinations is covered, some coefficients show strong correlations, e.g., $e_{25}-e_{14}$, $e_{35}-e_{13}$, and $e_{15}-e_{31}$. This is due to the fact that although a vast variety of geometries are generated, they originate from geometric transformations of 262 unique topologies. Since the piezoelectric properties are influenced by both the geometry and topologies of the unit cells, some correlation existing in the elementary topologies can influence the correlation in the whole dataset.

As mentioned before, dataset II is based on cubic unit cell volumes and thus only has five non-zero piezoelectric coefficients as the base material. However, due to the graph representation, a larger variety of topologies is obtained. This leads to a broader range of values of piezoelectric coefficients. Figure S5 shows a comparison of the five piezoelectric coefficients of both datasets. As can be seen from the pair plots, dataset I shows correlations between different coefficients with approximately triangular patterns in the distribution of lattices. On the other hand, dataset II has more arbitrary distributions of the datapoints with no obvious correlations between different coefficients. Moreover, while dataset I only shows positive values of all five coefficients, dataset II exhibits both positive and negative values of e_{31} and e_{32}.

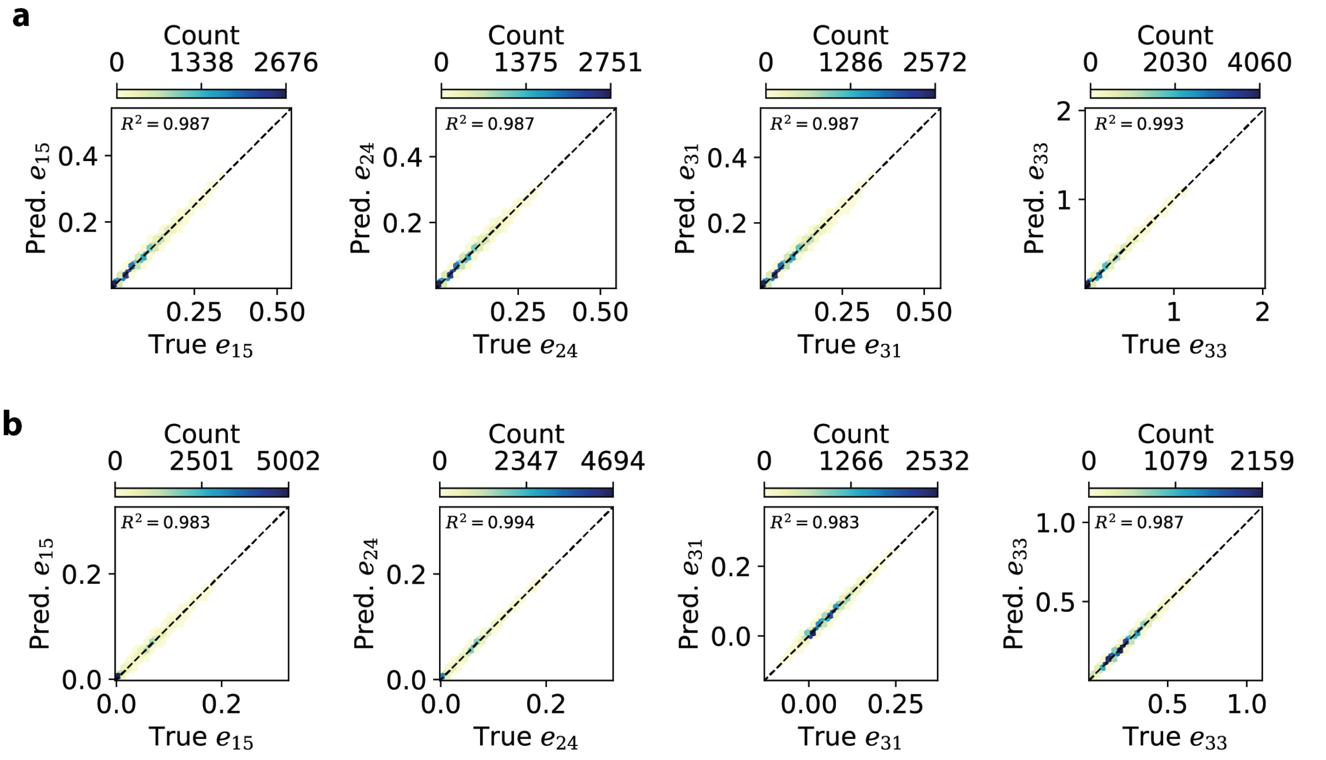

Figure S2. The parity plots for property prediction of datasets a. I and b. II. Here, any point lying on the unit slope line means a perfect match between predicted and true values.

4 Additional examples

In addition to the optimization examples shown in the main article, Figure S6 shows additional unit cells with exotic properties achieved through ML-based optimization. In the first example (Figure S6**a-c**), we show a lattice optimized for achieving unidirectional response in response to an applied electric field in direction-3. This is achieved by solving the following optimization problem:

$$\Theta^* \leftarrow \arg\max_{\Theta} J_1, \qquad \text{with} \quad \boldsymbol{e} = \mathscr{F}_\omega(\Theta) \quad \text{and}$$
$$J_1 = \left(e_{31} - e_{32}^2 - e_{33}^2\right) - \lambda_2 \left(\sum_{i=1}^{6} e_{1i}^2 + \sum_{i=1}^{6} e_{2i}^2 + \sum_{i=4}^{6} e_{3i}^2\right) \tag{6}$$

In the second example, as shown in Figure S6**d-f**, the unit cell design is optimized to achieve omnidirectional piezoelectricity or full anisotropy of the piezoelectric behavior by solving:

$$\Theta^* \leftarrow \arg\max_{\Theta} J_2, \qquad \text{with} \quad \boldsymbol{e} = \mathscr{F}_\omega(\Theta) \quad \text{and} \quad J_2 = \left(\sum_{i=1}^{3} \sum_{j=1}^{6} e_{ij}\right). \tag{7}$$

Figure S6**b** and **e** show the traces followed by the optimization algorithm for J_1 and J_2, respectively. Figure S6**c** and **f** show the piezoelectric surfaces (top) and the piezoelectric matrices (bottom) for these examples.

5 Principal component analysis (PCA)

Table S2. List of parameters used for the second ML frameworks' training and design optimization protocols.

Parameter	Notation	Value
ML framework #2 hyperparameters:		
Feature scaling	–	none, min-max-scaling
Connectivity encoder input dimension	–	278
Node position encoder input dimension	–	27
Connectivity encoder hidden dimensions	–	$512, 512, 512, 128$
Node position encoder hidden dimensions	–	$640, 640, 640, 512$
Connectivity decoder input dimension	–	$32 + 8$
Node position decoder input dimension	–	$32 + 8$
Connectivity decoder hidden dimensions	–	$128, 512, 512, 215$
Node position decoder hidden dimensions	–	$256, 512, 640, 640$
Connectivity decoder output dimensions	–	278
Node position decoder output dimensions	–	27
Property predictor hidden dimensions	–	$400, 800, 1000, 400, 400, 200$
Property Predictor output dimensions	–	5
Activation function hidden layer	–	ReLU
Connectivity (marg.) latent dimension	–	8
Node position (marg.) latent dimension	–	8
Overlapping latent dimension	–	32
Total latent dimension	–	48
Learning rate	–	0.0005
Batch size	–	8
Number of epochs	–	200
Optimizer	–	Adam[4]
Train/Test split	–	$0.97/0.03$
Optimization setup hyperparameters:		
Number of guesses	S	1
Number of random seeds for initial guess	Q	150
Number of epochs	E	30000
Optimizer learning rate	–	0.0001
Optimizer	–	Adam[4]

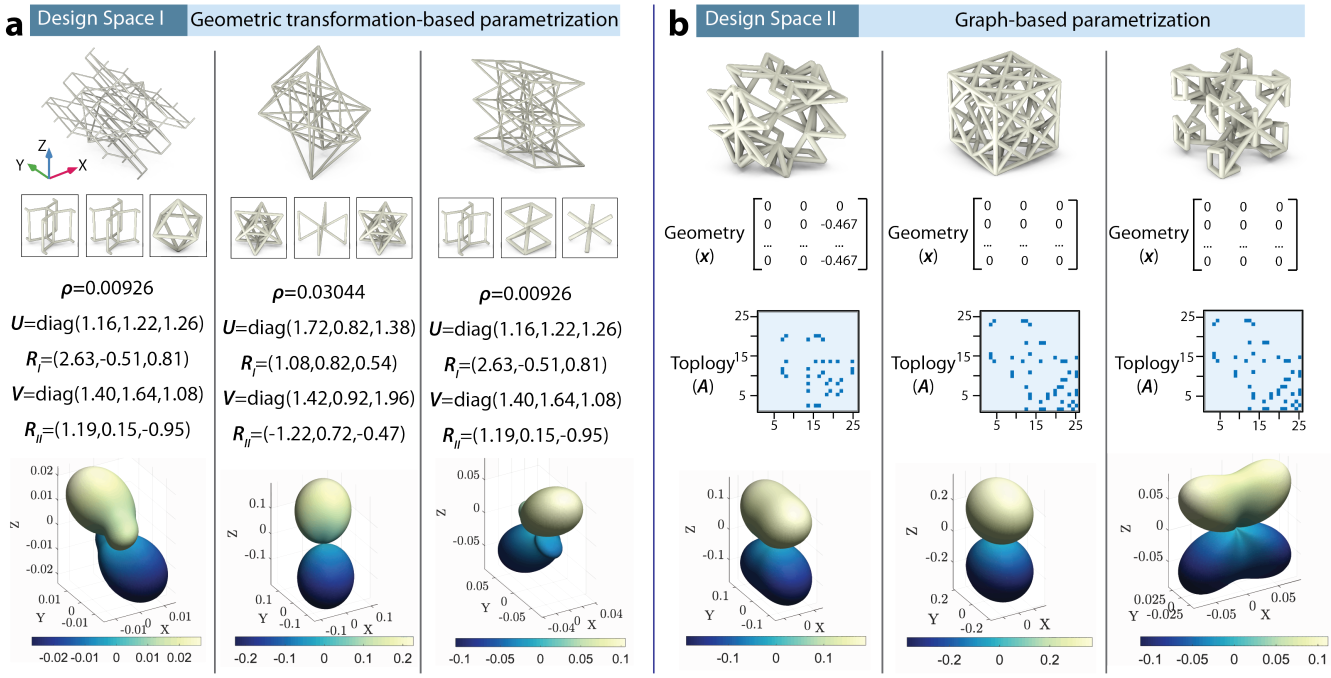

Figure S3. **Process for generating dataset from design space I and II. a.** Design space I based on the overlapping and affine transformations of seven primitive unit cells. Three example unit cells and their design parameters are shown along with their corresponding e_{33} surfaces. **b.** Design space II, based on the graph representation of unit cells. Three example unit cells are shown along with their geometric (in terms of the 27 nodal perturbations) and topological (connectivity matrix) parameters and corresponding e_{33} surfaces.

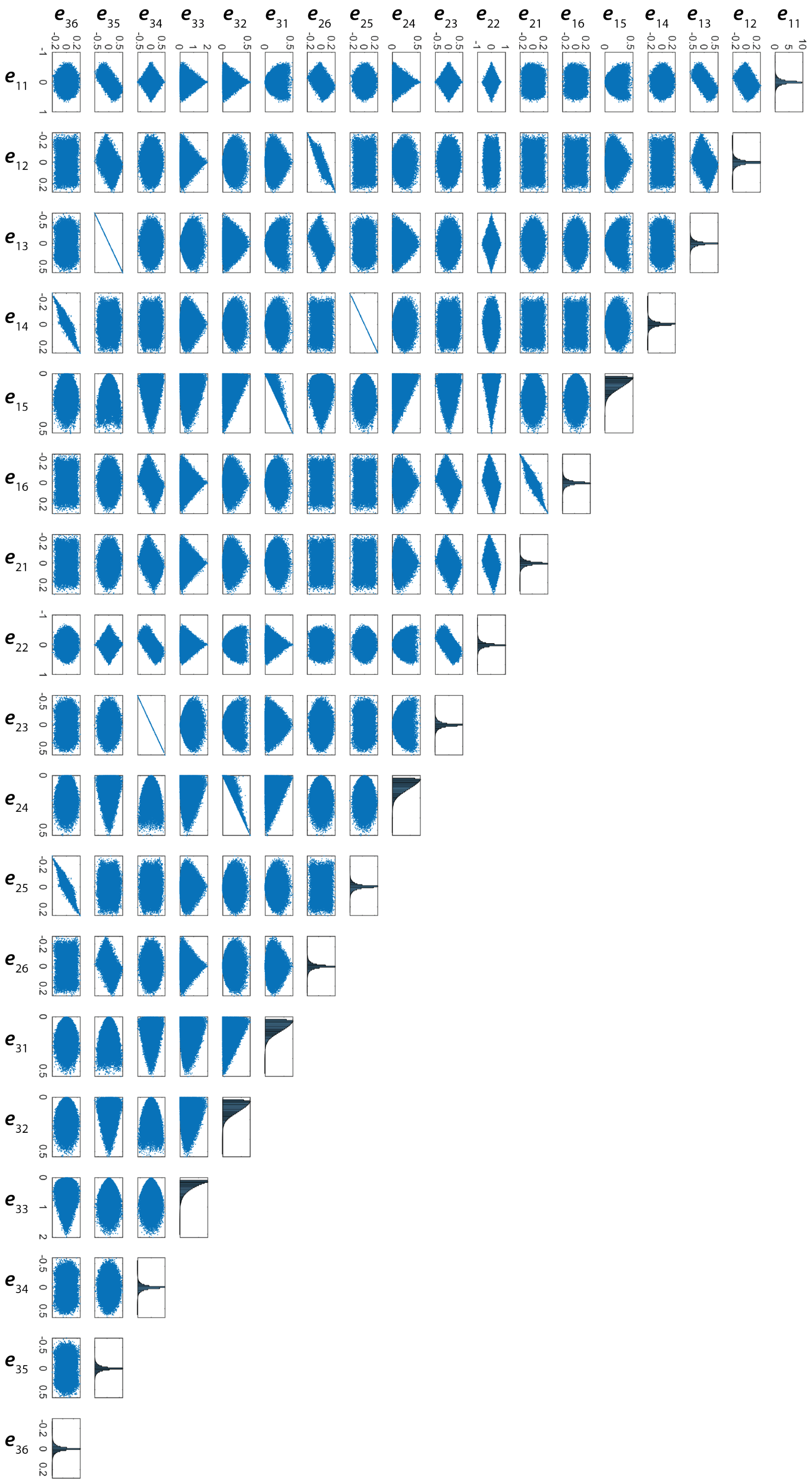

Figure S4. **Pair plots of the full piezoelectric tensor *e* for dataset I.** Each plot represents the distribution of all the unit cells in the 2D space of piezoelectric coefficients on its horizontal and vertical axes. A concentration of points along the diagonal, such as e_{13} vs e_{15}, of a plot represents a high correlation between the coefficients pertaining to the plot.

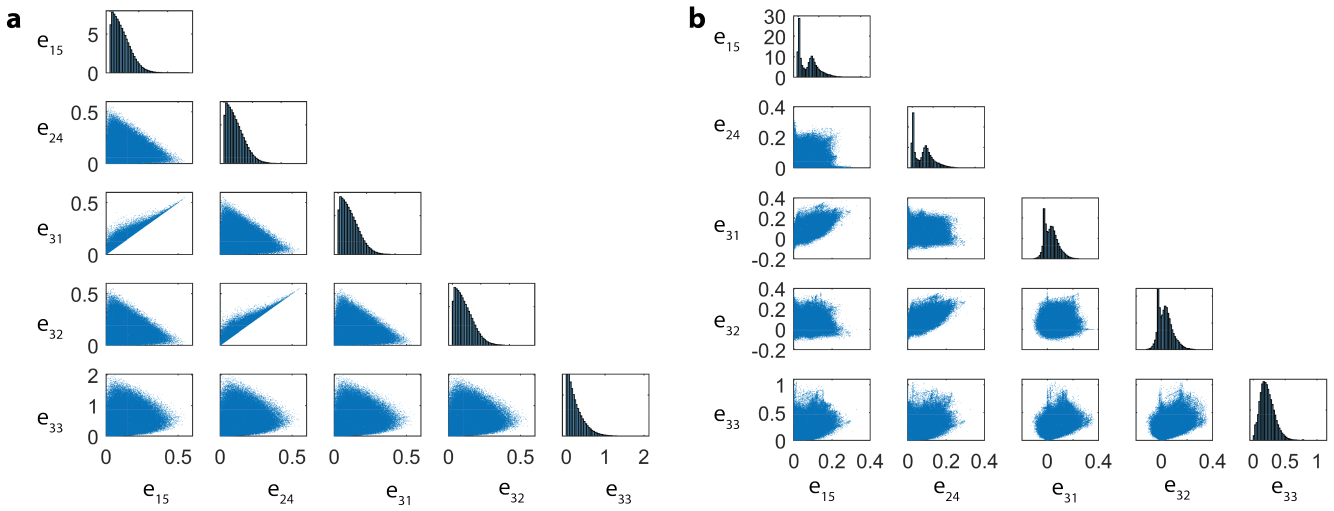

Figure S5. Pair plots of the five piezoelectric coefficients for a. dataset I, and **b.** dataset II.

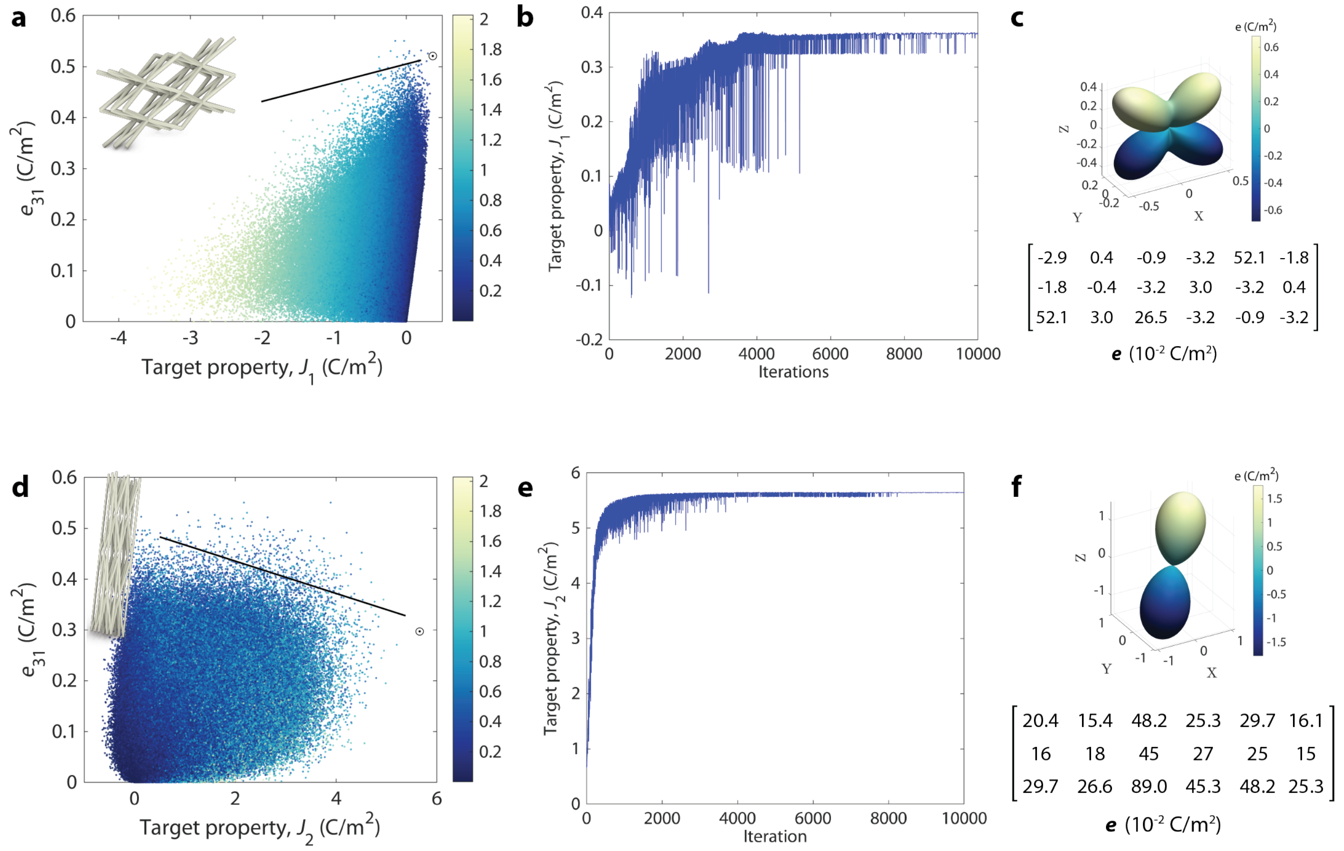

Figure S6. Additional examples of tailored piezoelectric responses achieved with the ML-based optimization. **a.** Unit cells with maximized J_1 for unidirectional piezoelectricity in the J_1 vs. e_{31} landscape. The unit cell lies outside the property space of the training dataset. **b.** The optimization trajectory to achieve the optimized design for J_1. **c.** The piezoelectric surface (top) and the piezoelectric matrix (bottom). **d.** Example of maximized J_2 for omnidirectional or fully anisotropic piezoelectricity, showing the optimized unit cell in the J_2 vs. e_{31} landscape. Similar to the first example, the optimized unit cell lies well beyond the training dataset. **e.** The optimization trajectory for achieving the optimized design for J_2. **f.** The piezoelectric response of this optimized unit cell.

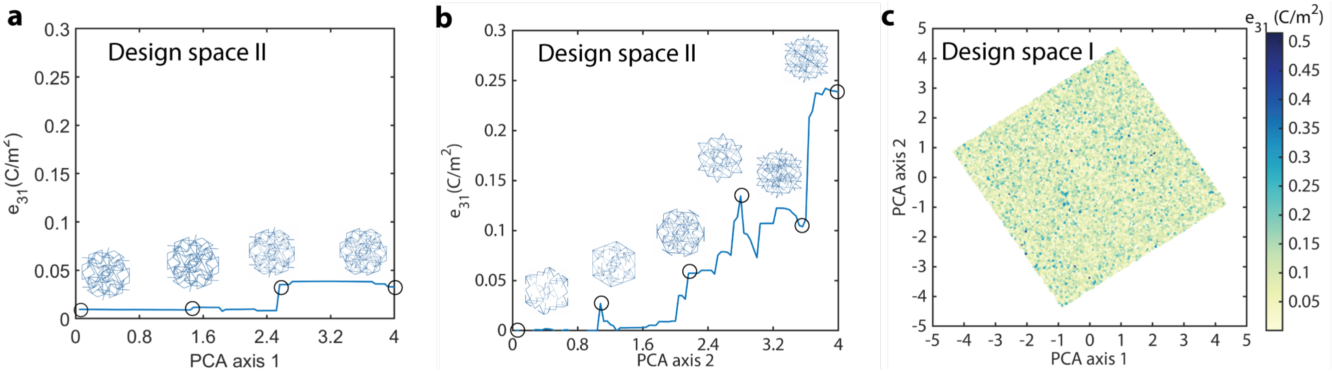

Figure S7. Principal component analysis of the two design spaces. For the design space II, **a.** shows the variation of e_{31} along the principal component axis 1, showing small variation in e_31, **b** variation of e_{31} along the principal component axis 2, with significant variations in the unit cell design as well as e_{31}. **c.** PCA of design space I in the parameter space. Due to the abrupt changes in piezoelectric properties with design parameters, variation of e_{31} does not exhibit a discernible trend.

References

1. Zhang, Y., Shang, S. & Liu, S. A novel implementation algorithm of asymptotic homogenization for predicting the effective coefficient of thermal expansion of periodic composite materials. *Acta Mech. Sinica* **33**, 368–381 (2017).
2. Yang, Q.-S. & Becker, W. Numerical investigation for stress, strain and energy homogenization of orthotropic composite with periodic microstructure and non-symmetric inclusions. *Comput. materials science* **31**, 169–180 (2004).
3. Lumpe, T. S. & Stankovic, T. Exploring the property space of periodic cellular structures based on crystal networks. *Proc. Natl. Acad. Sci.* **118**, e2003504118 (2021).
4. Kingma, D. P. Adam: A method for stochastic optimization. *arXiv preprint arXiv:1412.6980* (2014).
5. Zheng, L., Karapiperis, K., Kumar, S. & Kochmann, D. M. Unifying the design space and optimizing linear and nonlinear truss metamaterials by generative modeling. *Nat. Commun.* **14**, 7563 (2023).
